# EGFR inhibition for metastasized cutaneous squamous cell carcinoma in dystrophic epidermolysis bullosa

**DOI:** 10.1186/s13023-019-1262-7

**Published:** 2019-12-03

**Authors:** Andrea Diociaiuti, Holger Steinke, Alexander Nyström, Agnes Schwieger-Briel, Frank Meiss, Christina Pfannenberg, Leena Bruckner-Tuderman, Juri Ruf, Rita De Vito, May El Hachem, Dimitra Kiritsi

**Affiliations:** 1 Dermatology Unit, Bambino Gesù Children’s Hospital, IRCCS, Rome, Italy; 2 Department of Dermatology, Medical Center – University of Freiburg, Faculty of Medicine, University of Freiburg, Hauptstrasse 7, 79104 Freiburg, Germany; 3 Department of Paediatric Dermatology, University Children’s Hospital Zurich, 8091 Zurich, Switzerland; 4 Department of Radiology, University Hospital and Faculty of Medicine Tübingen, University of Tübingen, Tübingen, Germany; 5 Department of Nuclear Medicine, Medical Center – University of Freiburg, Faculty of Medicine, University of Freiburg, Freiburg, Germany; 6 Pathology Unit, Bambino Gesù Children’s Hospital, IRCCS, Rome, Italy

**Keywords:** Collagen VII, Skin fragility, Skin cancer, Cetuximab, Immunotherapy

## Abstract

Dystrophic epidermolysis bullosa (DEB) is a hereditary skin fragility disorder, characterized by trauma-induced blistering followed by soft tissue fibrosis. One of the most feared complications is the early development of aggressive cutaneous squamous cell carcinomas (SCC). For patients with locally advanced or metastasized SCCs treatment with cetuximab, a monoclonal antibody against epidermal growth factor receptor (EGFR), has been proposed and so far, treatment of five DEB patients with cetuximab has been published. With this report, we extend the spectrum of EB patients treated with cetuximab by adding two additional patients. Taking together all DEB cases treated with cetuximab, we propose that cetuximab should be administered as early as possible, since it seems to be more efficient and is accompanied by rather mild adverse effects. We also show that EGFR is frequently expressed in DEB-associated SCCs, although there were noticeable differences in the level of expression, which may influence responsiveness to EGFR-targeting therapies. Although only limited experiences with targeted cancer treatments in EB exist, such reports highlight the treatments’ effects in this specific cohort and assist our therapeutic decisions.

Dystrophic epidermolysis bullosa (DEB) is a hereditary skin fragility disorder, characterized by trauma-induced blistering followed by soft tissue fibrosis [1]. One of the most feared complications is the development of aggressive cutaneous squamous cell carcinomas (SCCs) [2, 3]. Patients suffering from the most severe generalized recessive DEB subtype (RDEB-gen sev) have the highest risk (rising up to 90.1% by the age of 55) [4]. Metastatic SCCs also represent the most common cause of death in adults with RDEB [4]. Moreover, although SCCs are usually well differentiated, they tend to relapse [5]. The standard treatment of EB-associated SCC is wide local excision. In some cases amputation is necessary [6]. Treatment with cetuximab, a monoclonal antibody against epidermal growth factor receptor (EGFR), has been proposed for patients with advanced or metastasized SCCs [6, 7]. Although not every determinant of cetuximab response or resistance has been identified, this treatment is associated with better responses, if EGFR is expressed in the tumor [8]. Besides the fact that no comprehensive studies on the expression of EGFR in EB-related SCCs have been published so far, EB patients with metastasized cutaneous SCCs treated with cetuximab are reported in the literature [8–10].

Here, we stained 10 RDEB-gen sev-associated SCCs of different differentiation grades for EGFR expression. The goal was to determine the percentage of patients, eligible for cetuximab treatment. In all EGFR stained positive, although there was considerable heterogeneity in the staining intensity (Fig. 1). Subsequently, we treated two patients with RDEB-gen sev with SCCs with cetuximab.

**Fig. 1:**
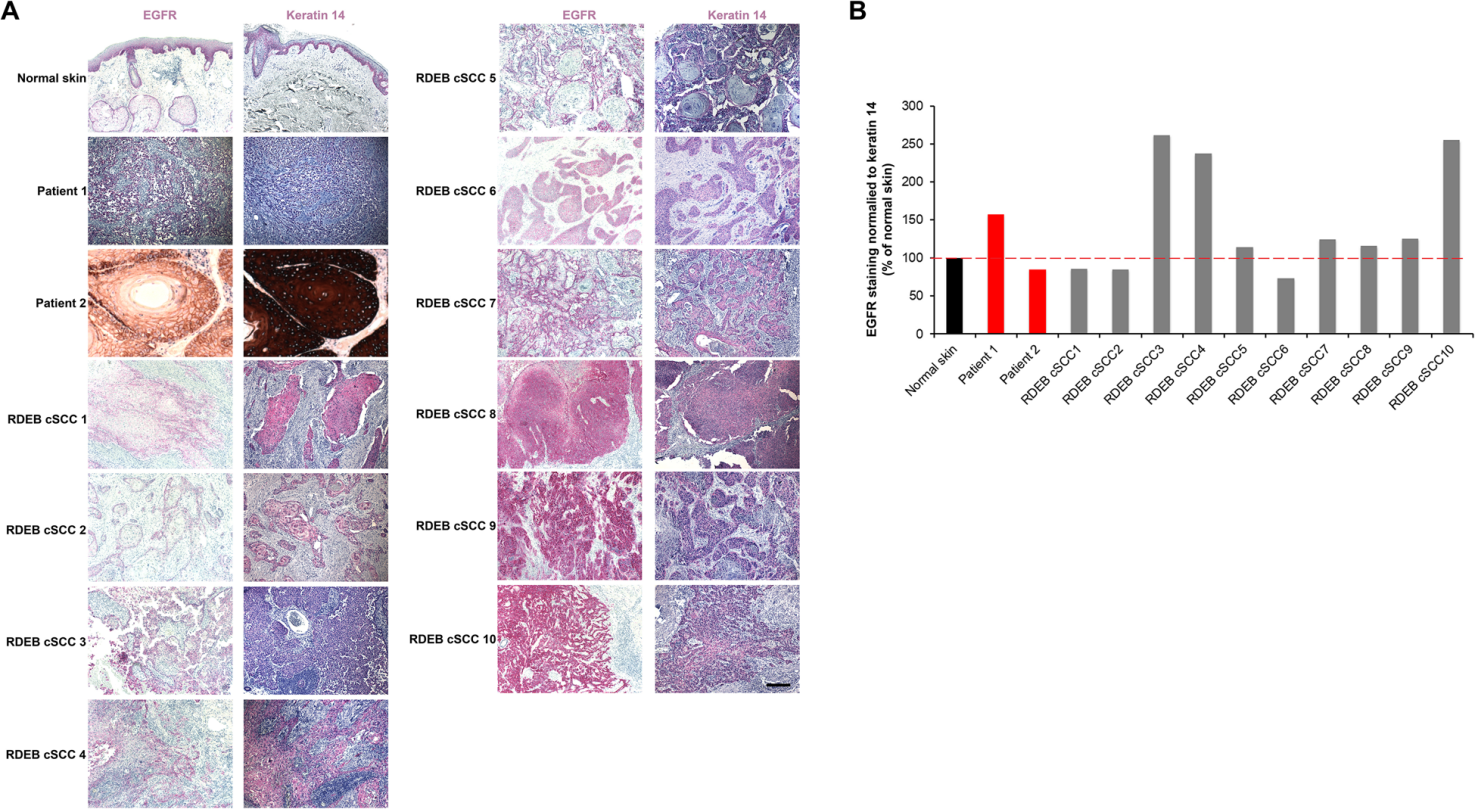
EGFR staining of 10 RDEB-related SCCs is positive in all tumors, although there is a notable variability among different tumors. **a** Ten primary RDEB-related cSCCs stained for EGFR and keratin 14. RDEB cSCCs are generally well differentiated and maintain keratin 14 expression [11]. **b** Quantification of the mean EGFR staining in tumor sections after normalization to keratin 14 staining in adjacent sections. The data are expressed as percentage of EGFR / keratin 14 ratio in control skin

Patient 1. A 49-year-old female with RDEB-gen sev with a history of multiple and multifocal cutaneous SCCs, predominately on the extremities, presented with a new large poorly differentiated SCC at the right lower leg (Fig. 2a). Wide local excision had initially been performed with histologically-proven clear peripheral and deep margins. Within 1 month after surgery the tumor recurred and grew rapidly. In addition, axillary and parailiacal lymph node metastases were detected by magnetic resonance imaging (MRI) and positron emission tomography / computed tomography (PET/CT-scan) with ^18^F-Fluoro-2-deoxy-2-D-glucose (FDG), which showed pathologically increased glucose metabolism (Fig. 2b). The patient refused lymph node dissection due to her severe skin condition and delayed wound healing. As the immunohistochemistry of the primary tumor from the right lower leg was positive for EGFR (Fig. 2c), the patient received cetuximab with a loading dose of 400 mg/m^2^ and afterwards 250 mg/m^2^ weekly for about 6 months. Around 5 months after cetuximab initiation, the parailiacal lymph node metastasis had almost disappeared and the axillary metastasis showed only minimal residual glucose hypermetabolism (Fig. 2b). During the treatment the patient experienced no adverse effects, beside slower wound healing. Unfortunately, the mass on the right lower leg did not regress. Six months after initiation of cetuximab therapy a haemodynamically-relevant bleeding of the tumor led to amputation of the right lower leg. Thereafter, the patient refused any further treatment or diagnostic procedures. Despite the fact that no more excisions, systemic treatments or radiotherapy were performed, the patient survived for 40 months after cetuximab discontinuation. This time period was longer than predicted on the basis of our experience and the data reported in literature with metastatic SCCs [3, 4].

**Fig. 2:**
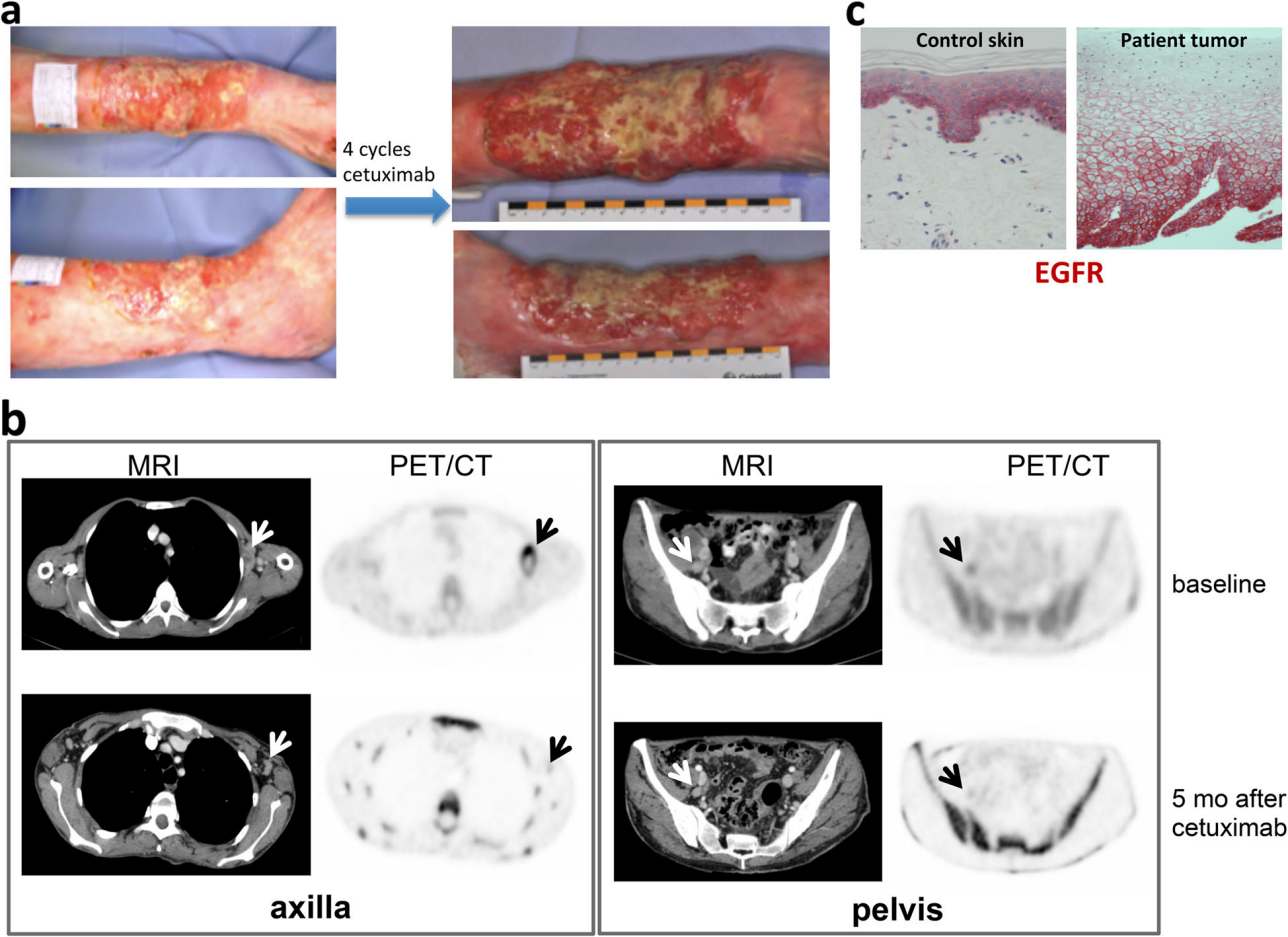
**a** Patient 1 presented with a cauliflower-like tumor at the right lower leg. The tumor did not regress after 4 cycles of cetuximab. **b** MRI and ^18^F-FGD-PET/CT at baseline showed hypermetabolic enlarged node metastases (white and black arrows, respectively) in the left axilla and pelvis (upper panel). Five months after cetuximab initiation a decrease of the axillary lymph node metastasis in size and metabolism was observed, while only a faint residuum was noticeable in the pelvic region (lower panel). Also note the increased bone marrow activity due to concomitant inflammatory reactions associated with the chronic wounds in EB. **c** IHC of the primary tumor of patient 1 revealed positive EGFR staining (red)

Patient 2. A 15-year-old girl with RDEB-gen sev presented with a large (> 15 cm) ulcerated skin tumor on the left deltoid (Fig. 3). She was in a poor general condition, displaying the characteristic RDEB-gen sev related clinical features. Histology confirmed a well differentiated SCC positive for EGFR (Fig. 3f**)**. CT scan revealed left axillary lymph nodes metastases. The deltoid SCC displayed initial regression after electrochemotherapy, but 1 month later it rapidly progressed with the onset of new nodular lesions on the sternum and enlargement of the primary tumor (Fig. 3a, c). A new total-body CT scan revealed multiple, new left clavicular and bilateral axillary lymph node metastases. Based on immunohistochemical analysis (Fig. 3f), cetuximab was administered with a weekly dosage of 250 mg/m^2^. After 3 months, the patient revealed reduced pain, the primary tumor (Fig. 3b) and the lesion on the back were flattened, while the sternal nodule continued to grow (Fig. 3d). After 24 weeks of treatment, the disease progressed with development of new nodules, increase of the previous lesions and pelvic pain. At this point, cetuximab was discontinued.

**Fig. 3:**
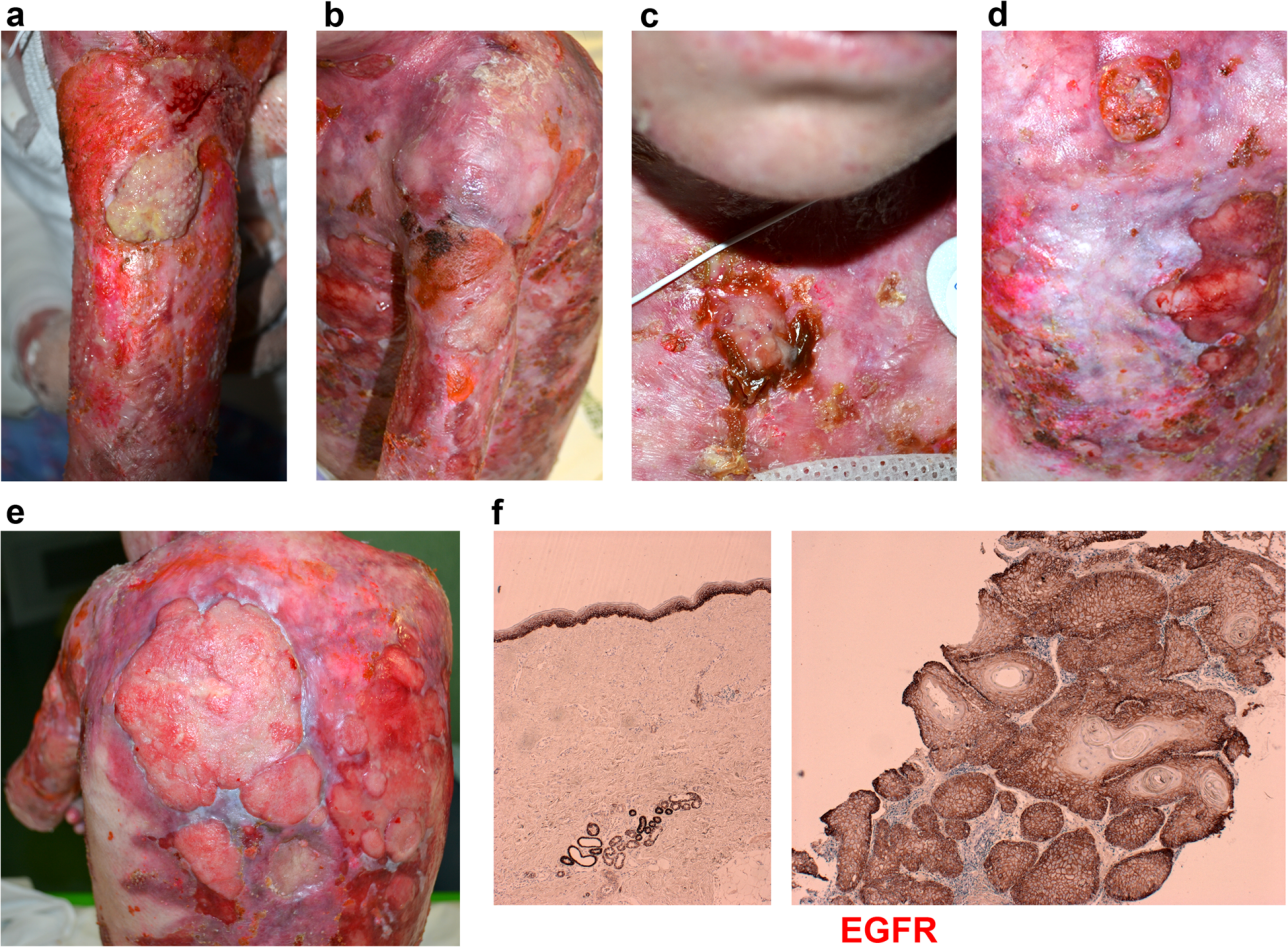
**a** SCC of the left deltoid region. **b** The same lesion flattened after 12 cycles of cetuximab. **c** Sternal nodular lesion of the SCC at initial presentation. **d** Increasing in size of the same lesion after 12 cycles of cetuximab. **e** Progression of the disease with onset of multiple new lesions of the dorsum. **f** IHC of the primary tumor of patient 2 revealed positive EGFR staining (brown)

With this report we extend the spectrum of EB patients treated with cetuximab, which is used in locally-advanced and metastasized head and neck SCCs [12]. In three previously published cases of EB [6, 8], cetuximab was administered sequentially after radio- and chemotherapy in order to reduce potential skin toxicity, bearing in mind that treated patients often develop papulopustular or acneiform rashes, xerosis, as well as alterations of hair and nail beds [13]. The already published EB patients died within a few months after cetuximab initiation (Table 1 and personal communication with Dr. Arnold in Basel, Switzerland). In another recently published case [10], cetuximab was given as a first-line treatment after diagnosis of lymph node metastasized SCC, with a progression-free survival of 9 months and a survival after treatment of at least 2 years. The treatment was well-tolerated, besides wound healing impairment. Our patient 1 showed a similarly long progression-free survival as in the aforementioned report and comparable adverse effects.

**Table 1:** EB patients treated with cetuximab in the literature

	Age at tumor’s presentation (years)	Tumor characteristics	Lymph node metastases present	Organ metastases present	Treatment options before cetuximab^a^	Patient outcome under cetuximab	Reference
1	29	primary tumor on right forearm; axillary lymph node metastases as well as in-transit cutaneous metastases on the right upper limb	axillary	–	–	treatment initially well-tolerated, but wound healing deficits after 6 months; progression free survival for 9 months; treatment discontinuation after 2 years after grade 2 allergic reaction; death 1 year later after use of other treatment options	Medek et al., J Dtsch Dermatol Ges. 2019
2	24	primary well-differentiated SCC on right elbow; subcutaneous metastasis next to SCC; tumor regrowth within weeks after 3x excisions (amputation declined by patient)	2 axillary	–	radiotherapy; chemotherapy with cisplatin and 5-fluorouracil (2 cycles)> > chemotherapy with cisplatin and paclitaxel for 1 month> > cetuximab for 12 cycles	besides acneiform folliculitis on the face treatment well-tolerated; progression free survival for 3 months	Arnold et al., Dermatology, 2009
3	26	moderately differentiated SCC on the dorsal right hand; quick tumor regrowth after excision, resulting in amputation	2/22 axillary	lung	axillary dis-section followed by local radiotherapy> > cetuximab for 10 cycles> > due to progression of lung metastasis combination with gemcitabine	treatment well tolerated; death around 3 weeks after initiation of cetuximab+ gemcitabine treatment on pneumonia	Kim et al., Br J Dermatol. 2013
4	43	well-differentiated SCC in axilla with extensive extranodal local spread; recurrence in axilla within 3 months after axillary node dissection	6/16 axillary	lung	axillary dis-section followed by local radiotherapy; cetuximab for 7 cycles> > due to development of multiple lung metastases: methotrexate 40 mg orally and then i.v. for 9 weeks> > treatment cessation due to progressive disease	initially development of a vesicular eruption, no other side effects; death 2 months after treatment discontinuation on pneumonia	Kim et al., Br J Dermatol. 2013
5	na	na	na	na	na	worsening of skin lesions	Maubec et al., J Clin Oncol. 2011

All patients published so far had severe generalized recessive DEB

Legend: +, present; −, absent; ^a^, treatment options are presented in chronological order; *na* not available

In our 2 patients with cutaneous SCCs and lymph node metastases, cetuximab was initially used as the sole treatment. Contrasting previous reports [8, 9], patient 1 survived for around 4 years after cetuximab initiation, while patient 2, who had a more advanced disease and poor general condition, survived only 4.5 months. In addition, pain improved in patient 2 during cetuximab treatment, allowing a better quality of life in absence of drug side effects. The different responses of the lymph node metastases versus the primary tumor, as shown in patient 1, might indicate intra-tumoral heterogeneity regarding the expression of EGFR and / or influences of the microenvironment on immunotherapy. Based on the overall survival, patient 1 had a better response to cetuximab than patient 2. This might at least partially be attributed to the higher EGFR / keratin 14 ratio in patient 1, as shown in Fig. 1.

We show that RDEB-gen sev–associated SCCs frequently express EGFR, although there were noticeable differences in the level of expression, which may influence responsiveness to EGFR-targeting therapies [14]. Together with the absence of major side effects as published so far, cetuximab could be indicated for this patient population with inoperable SCCs. Indeed, cetuximab mediates antibody-dependent cell-mediated cytotoxicity in the tumor and promotes cellular immunity. In order to counteract regulatory immunosuppressive feedback, it has been recently suggested to combine immune checkpoint inhibitors with cetuximab, to promote the adaptive and innate immunity against the tumor [15]. Recently, rigosertib has also been proposed from nonclinical studies as a therapeutic option for late stage, metastatic or unresectable RDEB SCCs. This molecule inhibits multiple signaling pathways with a similar profile to PLK1 and induces apoptosis in RDEB SCC keratinocytes [16].

Based on our experience, we hypothesize that cetuximab may be more efficient for metastasized RDEB-gen sev–associated SCCs, when administered early. In fact, reports published so far also suggest that efficacy might be higher if early administered, giving the patient a better chance of survival. Cetuximab could also improve quality of life in patients who cannot undergo other specific therapies. More studies should definitely be performed to confirm this hypothesis. Since only limited experiences with targeted cancer treatments in EB exist, such reports highlight the treatments’ effects in this specific patient cohort and assist our therapeutic decisions.

## Data Availability

Data sharing not applicable to this article as no datasets were generated or analysed during the current study.

## Abbreviations

DEB: Dystrophic epidermolysis bullosa
EB: Epidermolysis bullosa
EGFR: Epidermal growth factor receptor
IHC: Immunohistochemistry
MRI: Magnetic resonance imaging
PET/CT: Positron emission tomography / computed tomography
RDEB-gen sev: Severe generalized recessive dystrophic EB; SCC, squamous cell carcinoma
